# Subcellular compartmentation of sugar signaling: links among carbon cellular status, route of sucrolysis, sink-source allocation, and metabolic partitioning

**DOI:** 10.3389/fpls.2012.00306

**Published:** 2013-01-18

**Authors:** Axel Tiessen, Daniel Padilla-Chacon

**Affiliations:** Departamento de Ingenierïa Genética, CINVESTAV Unidad IrapuatoIrapuato, México

**Keywords:** sugar signaling, cell organelles, signal transduction, sucrose synthase, sucrose/hexose ratio, hexokinase, AGPase

## Abstract

Recent findings suggest that both subcellular compartmentation and route of sucrolysis are important for plant development, growth, and yield. Signaling effects are dependent on the tissue, cell type, and stage of development. Downstream effects also depend on the amount and localization of hexoses and disaccharides. All enzymes of sucrose metabolism (e.g., invertase, hexokinase, fructokinase, sucrose synthase, and sucrose 6-phosphate synthase) are not produced from single genes, but from paralog families in plant genomes. Each paralog has unique expression across plant organs and developmental stages. Multiple isoforms can be targeted to different cellular compartments (e.g., plastids, mitochondria, nuclei, and cytosol). Many of the key enzymes are regulated by post-transcriptional modifications and associate in multimeric protein complexes. Some isoforms have regulatory functions, either in addition to or in replacement of their catalytic activity. This explains why some isozymes are not redundant, but also complicates elucidation of their specific involvement in sugar signaling. The subcellular compartmentation of sucrose metabolism forces refinement of some of the paradigms of sugar signaling during physiological processes. For example, the catalytic and signaling functions of diverse paralogs needs to be more carefully analyzed in the context of post-genomic biology. It is important to note that it is the differential localization of both the sugars themselves as well as the sugar-metabolizing enzymes that ultimately led to sugar signaling. We conclude that a combination of subcellular complexity and gene duplication/subfunctionalization gave rise to sugar signaling as a regulatory mechanism in plant cells.

## INTRODUCTION

Through this review, we show how one should consider both extensive gene duplication and extensive cellular compartmentation of plant metabolism to fully understand sugar signaling. Establishment of sugar gradients across different subcellular compartments, cells, and organs is a central issue of plant physiology; therefore, we address the multiplicity of sucrolytic pathways in sink and source tissues.

## SUBCELLULAR COMPARTMENTATION

Eukaryotic organisms differ from prokaryotic organisms in that their metabolic activity occurs in different parts of the cell (Lunn, 2007). Every cellular compartment depends, to some extent, on other subcompartments for the supply and/or delivery of precursors and/or intermediates (Stitt, 1997). Since primary pathways occur in different organelles, one should assume *a priori* that sugar perception and signal transduction is also compartmentalized (Lunn, 2007); however, most research on plant metabolomics and sugar signaling has ignored such a premise. In *Arabidopsis*, bulk tissue (from either rosette plants or germinating seedlings) is typically harvested and homogenized to determine metabolite levels as a bulk megacompartment, thereby both ignoring diversity of cell types and further mixing subcellular organelles (Osuna et al., 2007; Sulpice et al., 2009). Therefore, some mutants of key enzymes of sucrose metabolism have sometimes been reported to have no “obvious” phenotype (Bieniawska et al., 2007). To advance sugar signaling research, one should use a diverse array of experimental approaches for determining developmental gradients and subcellular levels of many biomolecules (Geigenberger et al., 2011; Kueger et al., 2012).

## SPATIAL DISTRIBUTION OF PROTEINS AND RNA

Plant gene expression patterns and protein location have been traditionally analyzed with β-glucoronidase, luciferase, and green fluorescent protein (Hejatko et al., 2006; Lalonde et al., 2008). Many constructs can be transiently expressed with new methods (Li et al., 2009), but experimental elucidation of protein location must be done in carefully sectioned tissues with laser-capture micro-dissection (Chang et al., 2012). Localization of mRNA is mostly determined with *in situ* hybridization (Borisjuk et al., 2005; Fallahi et al., 2008). In the post-genomic era, bioinformatic prediction of protein targeting is more extensively used and could eventually replace some experimental approaches (Gomez-Anduro et al., 2011).

## SPATIAL DISTRIBUTION OF METABOLITES

The location of either mRNA or proteins reveals biosynthetic potential, but it is not necessarily where the metabolite finally accumulates (Lee et al., 2012). Metabolic networks represent a completely different level of realization of genomic information that is not always correlated with proteins and nucleic acids (Saito and Matsuda, 2010; Kueger et al., 2012).

Microscopy is the standard method for determining the location of biomolecules in plant organs because molecular gradients produce different colors and intensities in specific cells. Microscopy generates qualitative information, but unfortunately it has not yet been adapted for quantitative measurement of metabolites and enzymes.

Non-aqueous fractionation (NAF) is a powerful technique for separating subcellular compartments under conditions in which biological activities are completely arrested (Farre et al., 2001; Geigenberger et al., 2011). This method allows to calculate *in vivo *mass-action ratios of all reactions of sucrose metabolism (Tiessen et al., 2002). Metabolomic NAF analysis in barley seeds (Tiessen et al., 2012), *Arabidopsis* leaves (Fettke et al., 2005a; Geigenberger et al., 2011), potato leaves (Fettke et al., 2005b), and potato tubers (Farré et al., 2006, 2008, 2001), shows marked differences in compartmentation. The classical assumptions about metabolite subcellular distribution are not always true in all species and in all organs. The subcellular ADPGlc level in barley mutants, for example, provides important clues about metabolic regulation in cereal endosperms (Tiessen et al., 2012).

Improved methods for single-cell transcriptomics, proteomics, and metabolomics are needed for a holistic understanding of sugar signaling (Dai and Chen, 2012). Fluorescence techniques reveal dynamics and localizations of molecular interactions within cells (Lalonde et al., 2005); mechanical- and affinity-based technologies are used to isolate and analyze individual cell types in plants (Wang and Ruan, 2012); and system-level analyses of specific cell types in plants may soon become standard (Kueger et al., 2012).

## METABOLISM AND REGULATION: EXAMPLES OF A DUAL ROLE

**FIGURE 1:**
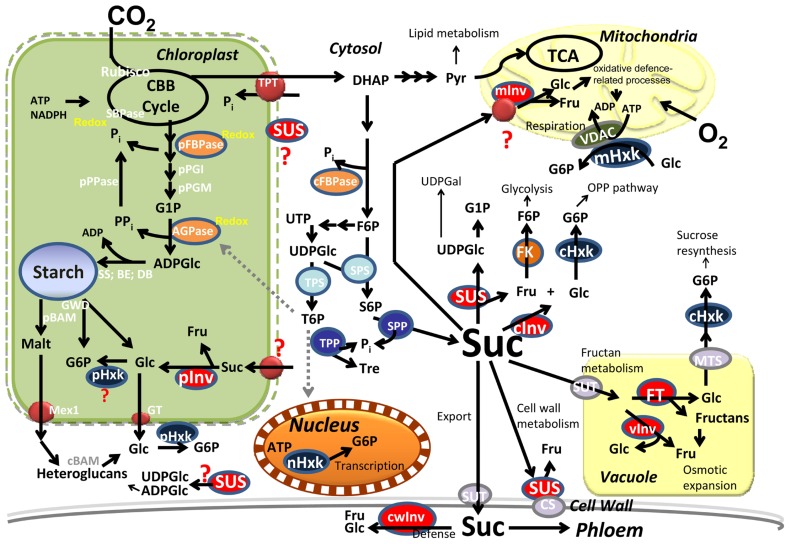
**Central metabolism in photosynthetic cells**. Carbon is converted into starch in the plastid and sucrose (Suc), in the cytosol. Suc is partitioned into different pathways by multiple isozymes in the different subcellular compartments. Signaling metabolites (Pi, T6P) coordinate fluxes within the cytosol and the plastids. BE, branching enzyme; cBAM, pBAM, cytosolic and plastidial beta-amylase; CBB; Calvin–Benson–Bassham; CS, cellulose synthase; DB, debranching enzyme; DHAP; dihydroxyacetone phosphate; Fru, fructose; cFBPase, pFBPase, cytosolic and plastidial Fructose-1,6-bisphosphate phosphatase; FK, fructokinase; FT, fructosyl-transferase; Glu, glucose; G6P, glucose-6P; G1P, glucose-1P; GT, glucose transporter; GWD, glucan water dikinase; cHxk, mHxk, nHxk, pHxk, cytosolic, mitochondrial, nuclear, and plastidial hexokinase; cInv, cwInv, pInv, vInv, cytosolic, cell wall, plastidial, and vacuolar invertase; Malt, maltose; Mex1, maltose exporter; Pi, inorganic phosphate; PPi, pyrophosphate; Pyr, pyruvate; S6P, sucrose-6-P; SBPase, sedoheptulose-1,7-bisphosphate phosphatase; SPP, sucrose-6-P phosphatase; SPS, sucrose-6-P synthase; SS, starch synthase; SUS, sucrose synthase; SUT, sucrose transporter; Tre, trehalose; T6P, trehalose-6-phosphate; TCA, tricarboxylic acid cycle; TPP, trehalose-6-P phosphatase; TPT, triose-P translocator; VDAC, voltage-dependent anion channel.

Neither compartments nor functions should be mixed. For some proteins, there is a risk of confusing metabolic activity with signal perception. Few metabolic enzymes “moonlight” as transcriptional regulators. The best known example in plants is hexokinase (HXK; (Harrington and Bush, 2003). In addition to catalyzing the first step of glycolysis, HXK is also a glucose sensor (Moore et al., 2003; Rolland et al., 2006) and in plants it transduces downstream signals, both transcriptionally (Baena-Gonzalez et al., 2007) and post-transcriptionally (Tiessen et al., 2003). Plant HXKs are encoded by a family of 5–10 genes (Claeyssen and Rivoal, 2007) which can be active in several compartments (Balasubramanian et al., 2007; Damari-Weissler et al., 2007; **Figure 1**). AtHXK1 is predominantly associated with the mitochondria but can also occur in the nucleus (Cho et al., 2009), where it associates with transcriptional complexes (Cho et al., 2006) to regulate gene expression (Balasubramanian et al., 2007). This remarkable bi-functionality enables crosstalk between compartments and metabolic pathways.

Some HXKs have similar catalytic activity, but they are not interchangeable for the regulatory function (Rolland et al., 2006). Other isozymes might have lost their original biochemical function during endosymbiotic evolution. In *Arabidopsis*, three of six HXK paralogs lack catalytic activity (Cho et al., 2006; Karve et al., 2008). These hexokinase-like (HKL) proteins have been experimentally detected in the mitochondria (Heazlewood et al., 2007) and can cause an unusual root hair phenotype (Karve et al., 2010).

The existence of HXK-based sugar signaling was initially questioned because it was isoform-specific and further because the glucose phenotype was only observed at specific stages of germination (Halford et al., 1999). Later it was found that two signaling pathways involving both SnRK1 and HXK regulate key enzymes of sucrose-starch metabolism (Tiessen et al., 2003; McKibbin et al., 2006). We believe the importance of such key enzymes should be re-evaluated in more detail and separately for each isoform, organelle, stage of development, and species.

## DO SOME SUGAR TRANSPORTERS ALSO ACT AS SENSORS?

The perception of metabolites can occur at the plasmatic membrane, as the first site of sugar signaling (Lalonde et al., 1999). H^+^-sucrose co-transporters (e.g., SUC2 and SUT1) are crucial for sucrose loading into the phloem, but their role as sensors remains elusive. In yeast, the glucose transporters RGT2 and SNF3 act as low- and high-affinity glucose sensors, respectively (Ozcan et al., 1996; Belinchón and Gancedo, 2007). An additional sensing mechanism involves RGS1, a negative regulator of G-protein signaling (Chen et al., 2003; Urano et al., 2012). In *Arabidopsis*, however, regulatory proteins are believed to be absent. Ectopic overexpression of the hexose transporter 2 gene alters glucose sensing and suggests that post-germinative development not only depends on cytosolic AtHXK1 but also on the entry of sugars through the membrane (Padilla-Chacon et al., 2010).

## METABOLITE COORDINATION BETWEEN CYTOSOL AND PLASTIDS

Metabolism of trehalose-6-phosphate (T6P) is needed for reproductive development in maize (Satoh-Nagasawa et al., 2006) and *Arabidopsis* (Eastmond et al., 2002), and is also important for seed germination (Gomez et al., 2006) and sugar signaling (Kolbe et al., 2005). T6P is synthesized in the cytosol and is strongly correlated with sucrose levels and the rate of starch synthesis in the plastid (Lunn et al., 2006). Supplying T6P to isolated chloroplasts promotes redox activation of AGPase (Kolbe et al., 2005), although the molecular mechanism is not fully understood. The direct effects of T6P on signaling/target proteins still need to be characterized in detail. T6P may act allosterically through either SnRK1 or NADP-thiorredoxin reductase C, which regulates AGPase (Michalska et al., 2009). T6P may act as a secondary messenger of carbon status between the cytosol and the chloroplasts (Kolbe et al., 2005).

## METABOLIC ROUTES FOR SUGAR SIGNALING

Overexpression of yeast invertase (INV) and bacterial glucokinase in the cytosol was intended as a strategy to increase sucrose import and sink strength in potato tubers (Trethewey et al., 1998). Contrary to expectation, a futile cycle of sucrose degradation and resynthesis is created, leading to decreased starch content in the transgenic lines (Trethewey et al., 1999).

Heterologous expression of sucrose phosphorylase (SuPho) was used to bypass SUS (sucrose synthase)/fructokinase (FK) and INV/HXK routes (Fernie et al., 2002). It decreases cytosolic sucrose levels (Tiessen et al., 2002). Reduction of starch in the SuPho transformants occurred from an ~40% decrease in AGPase activity and the redox activation state (Tiessen, 2002). Ectopic overexpression of either INV or SuPho affects the internal oxygen levels in growing tubers and is correlated with decreased starch content (Bologa et al., 2003). The INV/HXK pathway is, therefore, more energy demanding, and the SUS/FK pathway allows to maintain a higher cellular energy state under low-oxygen conditions (Bologa et al., 2003).

The different routes of sucrose degradation are not interchangeable because the subcellular levels of hexoses and sucrose produce different signals that activate different metabolic pathways (**Figure 1**). A low sucrose/hexose ratio promotes respiration over starch synthesis (Geiger et al., 1998). Expressing INV in either the apoplast or the cytosol leads to very different results (Farre et al., 2008; Ferreira and Sonnewald, 2012). Inhibition of SUS decreases specifically starch accumulation but not protein or lipid synthesis (Zrenner et al., 1995; Angeles-Núñez and Tiessen, 2010).

## MULTIPLE SUCROLYTIC ROUTES

Sucrolytic INV activity is critical for metabolism in plants (Ferreira and Sonnewald, 2012), and it might also have a direct role in signaling (Roitsch, 1999; Farre et al., 2008). *Arabidopsis* has six cell wall INV, two vacuolar INV, and 11 neutral/alkaline INV genes (Sherson et al., 2003). The functional roles of different INV isozymes are still not understood because subcellular location affects the different hexose pools that may be independently sensed (Ji et al., 2005; Xiang et al., 2011; **Figure 1**). Neutral/alkaline INVs have ascribed functions for energy metabolism and oxidative stress in the mitochondria (Xiang et al., 2011; Martïn et al., 2012), for photosynthesis in plastids (Murayama and Handa, 2007), and for overall cell development (Welham et al., 2009). Cytosolic, neutral INVs interact with phosphatidylinositol monophosphate 5-kinase and regulates root cell elongation (Lou et al., 2007). Cell wall INVs have been associated with carbon partitioning (Roitsch et al., 2003) and regulation of cellulose genes in cotton (Yang et al., 2008). Vacuolar INVs are needed in cell expansion, via osmotic-dependent and -independent pathways (Wang et al., 2010). The differential expression patterns of cwINV and vINV have also provided insight into early seed development (Wang and Ruan, 2012).

Sucrose synthase is important for primary metabolism (Geigenberger and Stitt, 1991), and has been implicated in long-distance carbon allocations, stress responses, and symbiotic interactions (Koch, 2004; Barratt et al., 2009; Coleman et al., 2009). SUS preferentially partitions carbon toward starch, in both potato tubers (Zrenner et al., 1995) and *Arabidopsis* seeds (Angeles-Núñez and Tiessen, 2010). This likely occurs through provision of direct or indirect intermediates of starch or cellulose synthesis (Fujii et al., 2010; Cai et al., 2011). The expression patterns of SUS isoforms, which have different subcellular locations, suggests specific signaling functions (Brill et al., 2011).

The *Arabidopsis* genome contains six SUS genes whose exact function remains to be known, since most mutants show few observable effects (Bieniawska et al., 2007) or present only subtle metabolic phenotypes in specific tissues and developmental stages (Angeles-Núñez and Tiessen, 2010, 2012). AtSUS2 appears to be specifically expressed in seeds and is not induced in leaves in response to either abiotic stresses or sugar feeding (Baud et al., 2004; Núñez et al., 2008). Mutants of SUS paralogs have not been analyzed with subcellular methods, but the pleitropic effects on metabolite homeostasis (Angeles-Núñez and Tiessen, 2010) cannot be simply explained by a catalytic function. Elucidating the structure of SUS isoforms could provide further insights into the signaling mechanisms and regulatory interactions that occur with other cellular targets (Zheng et al., 2011).

## SUCROSE 6-PHOSPHATE SYNTHASE

Sucrose 6-phosphate synthase (SPSs) are encoded by a multigene family whose tissue- and developmental stage-specific expression patterns appear to have functional significance (Salerno and Curatti, 2003). The activities of both SPS and sucrose 6-phosphate phosphatase (SPP) are required for sucrose metabolism (Lunn, 2002; Salerno and Curatti, 2003), but have not yet been directly implicated in sugar signaling. Nevertheless, the similarity between S6P and T6P suggests a sugar signaling role for either sucrose or trehalose metabolism (**Figure 1**).

## POST-GENOMIC RESEARCH

Genome-scale metabolic modeling is well-established for microbes (which typically have only one enzyme per reaction in the cytosol), but is not yet established in plants, which posses many different isozymes and subcellular compartments (Caspi et al., 2008). Various databases contain lists of reactions and putative metabolic pathways that are based on automatic gene annotations via BLAST (Youens-Clark et al., 2011; Kanehisa et al., 2012). The pathways in MetaCyc are manually curated (Caspi et al., 2012); however, metabolic models neither incorporate paralog nor subcellular information (Fernie and Stitt, 2012). Another difficulty for plants occurs with an assignment of catalytic function that is only based on overall sequence similarity. Multiple isozymes of HXK, SUS, or INV might share the similar folding that is needed for binding of metabolites and may be involved in signaling functions, but not catalysis (Karve et al., 2008).

## CONCLUSION

Sugar signaling research has advanced rapidly in recent years. Learning more will require acknowledging the importance of subcellular compartmentation and routinely implementing all available methods (Kueger et al., 2012). A key feature in signaling is that sucrose, for example, does not freely cross cell membranes. Thus it was the combination of subcellular organization plus the recruitment of potential signaling molecules that are differentially membrane permeable (plus gene duplication/specialization) that drove the occurrence of signaling.

Since carbon metabolism occurs simultaneously in different organelles, different sensors may be required under specific conditions or circumstances. Specific isoforms of sucrolytic enzymes are therefore not redundant but complementary for signaling or catalysis. We propose that different sucrolytic enzymes are important for channeling carbon into different metabolic routes, and we further postulate that sugar signals are specific for each paralog/pathway.

## Conflict of Interest Statement

The authors declare that the research was conducted in the absence of any commercial or financial relationships that could be construed as a potential conflict of interest.
